# Single-cell RNA sequencing of anaplastic ependymoma and H3K27M-mutant diffuse midline glioma

**DOI:** 10.1186/s12883-024-03558-7

**Published:** 2024-02-21

**Authors:** Dongdong Zang, Zilong Dong, Yuecheng Liu, Qian Chen

**Affiliations:** Department of Neurosurgery, Shenzhen Children’s Hospital, 7019 Yitian Road, Futian District, Shenzhen, Guangdong China

**Keywords:** Anaplastic ependymoma, H3K27M-mutant diffuse midline glioma, Single-cell RNA-sequencing, Intratumor heterogeneity

## Abstract

**Background:**

Anaplastic ependymoma and H3K27M-mutant diffuse midline glioma are two common subtypes of brain tumors with poor long-term prognosis. The present study analyzed and compared the differences in cell types between two tumors by single-cell RNA sequencing (scRNA-seq) technology.

**Methods:**

ScRNA-seq was performed to profile cells from cancer tissue from anaplastic ependymoma patient and H3K27M-mutant diffuse midline glioma patient. Cell clustering, marker gene identification, cell type annotation, copy number variation analysis and function analysis of differentially expressed genes were then performed.

**Results:**

A total of 11,219 cells were obtained from anaplastic ependymoma and H3K27M mutant diffuse midline glioma, and these cells categorized into 12 distinct clusters. Each cell cluster could be characterized with specific cell markers to indicate cellular heterogeneity. Five cell types were annotated in each sample, including astrocyte, oligodendrocytes, microglial cell, neural progenitor cell and immune cell. The cluster types and proportion of cell types were not consistent between the two brain tumors. Functional analyses suggest that these cell clusters are involved in tumor-associated pathways, with slight differences in the cells of origin between the two tumors. In addition, cell communication analysis showed that the NRG3-ERBB4 pair is a key Ligand-receptor pair for anaplastic ependymoma, while in H3K27M-mutant diffuse midline glioma it is the PTN-PTPRZ1 pair that establishes contact with other cells.

**Conclusion:**

There was intratumor heterogeneity in anaplastic ependymoma and H3K27M mutant diffuse midline glioma, and that the subtype differences may be due to differences in the origin of the cells.

**Supplementary Information:**

The online version contains supplementary material available at 10.1186/s12883-024-03558-7.

## Introduction

Brain tumors are the leading cause of cancer-related deaths in children [1]. Although advances in surgery and adjuvant therapy have improved survival of children with medulloblastoma and low-grade glioma (LGG), whose 5-year survival rate is now over 75% [2, 3], the prognosis for other tumors such as anaplastic ependymoma and diffuse midline glioma (DMG) continue to have a poor prognosis.

Ependymal tumors (EPN) is a common pediatric malignancy, a tumor of the central nervous system tumors of ependymal cells in the ventricles and central canal of the spinal cord or nests of ependymal cells in the white matter of the brain, accounting for death in approximately 45% of affected individuals [3–5]. Anaplastic ependymoma is a WHO grade III tumor composed of poorly differentiated ependymal cells with active mitosis, often accompanied by microvascular proliferation and tumor necrosis [6, 7]. The progression-free survival (PFS) and overall survival (OS) of patients with anaplastic ependymoma were significantly lower than those of patients with other types of ependymoma [8]. Brain cancers are usually treated with standard therapy consisting of extensive surgical resection followed by localized radiation therapy, but this may can cause complications in the developing brain. At present, there are no reports confirming the efficacy of chemotherapeutic drugs for these tumors [9, 10].

H3K27M-mutant diffuse midline glioma (BG) is a malignant tumor of the central nervous system, ranked as WHO grade IV [7]. It is present in the spinal cord, brainstem, pineal region, and thalamus, and exhibits aggressive clinical behavior. BG is the second most common childhood malignant brain tumor [11]. H3K27 diffuse midline gliomas have a median overall survival of 9–12 months after diagnosis [12]. Current treatments include chemotherapy, radiotherapy, immunotherapy, and intratumoral targeted therapy, but do not extend life expectancy of patients and have poor prognosis [13].

Many transcriptomic studies have uncovered that the pathogenesis of EPN and BG can be attributed to abnormalities in key genes and signaling pathways, such as FGFR gene, PI3K-Akt-mTOR, IL6/STAT3 pathway [14–16]. However, these studies are dependent on bulk RNA-seq profiling, which limited their ability to analyze inter-tumor and intra-tumor heterogeneity. A deep understanding of heterogeneity and the interplay between tumor cells and their microenvironment could allow for the development of new therapeutic approaches for treating EPN and H3K27M-mutant diffuse midline glioma.

Single-cell RNA sequencing (scRNA-seq) has made it progressively possible to uncover the complex heterogeneity in tumor microenvironment, cellular diversity and intercellular communication [17–19]. Recently, scRNA-seq has been performed to dissect heterogeneity and cell subpopulations of many types of tumor, such as liver cancer [20, 21], lung cancer [22], breast cancer [23, 24]. Nonetheless, heterogeneity and cell communication at single-cell resolution in EPN and BG remains poorly understood. In order to further explore the development process and mechanism of these two tumors and provide data support for clinical diagnosis and treatment, a droplet-based scRNA-seq was used to profile single cells of EPN and BG.

## Materials and methods

### Sample collection

The present study was approved by the Research Ethics Committee of Shenzhen Children’s Hospital. The research was conducted with the informed consent of each participant, and all participants provided a signed informed consent. One anaplastic ependymoma (EPN) and one H3K27M mutant diffuse midline glioma (BG) sample were collected from Shenzhen Children’s Hospital, and the clinical information of the samples is shown in **Table **S1.

### Preparation of single-cell suspensions

Tumor samples were separated into single cell suspensions using a combination of mechanical dissociation and enzymatic degradation of the extracellular matrix. Enzymatic digestion of the samples was performed using a Tumor Dissociation Kit (Miltenyi Biotec, Gladbach, Germany) following the manufacturer’s instructions. In short, tissues were minced and mixed with 200 µL enzyme H, 25 µL enzyme A, 100 µL enzyme R, and 4.7 mL Dulbecco’s Modified Essential Medium and loaded into MACS C Tube (130-094-392, Miltenyi Biotec). It was then dissociated three times using mild MACS™ dissociation reagent (130-093-235, Miltenyi Biotec) and the samples were incubated at 37 °C for 30 min between each dissociation step. Large particles, erythrocytes, and dead cells were removed using a filter (40 μm), Red Blood Cell Lysis Solution (130-094-183, Miltenyi Biotec), and Dead Cell Removal Kit (Miltenyi Biotec), respectively.

### Single-cell RNA sequencing

A Chromium Single Cell 3′ Reagent kit (10× Genomics, Pleasanton, CA, USA) was used to create scRNA-seq libraries. The suspension of single cells was loaded into the Chromium Single Cell Controller Instrument (10× Genomics) to generate Gel Beads-in-emulsion (GEMs). Then, barcoded full-length cDNA was then used for the reverse transcription reactions, followed by the disruption of emulsions using the recovery agent. DynaBeadsMyone Silane Beads (Thermo Fisher Scientific, Waltham, MA, USA) were used to clean the cDNA. Subsequently, the amplified cDNA was fragmented, end-repaired, A-tailed, index adapter ligated, and library amplified. Qubit dsDNA HS Assay Kit (Q32854, Agilent, USA) was used for library quantification, and the standard of charge was about 400–500 bp for the main peak. Libraries were sequenced on an Illumina sequencing platform (HiSeq X Ten; Illumina, USA).

### 10x Genomics scRNA-seq data analysis

#### Data comparison and statistics

The official 10× software CellRanger (V6.1.1) was used to filter, align, and map raw library reads for analysis [25]. The reads were aligned to the reference genome (GRCh38-2020-A) using STAR [26] alignment software. After alignment, the expression of each gene in each cell was obtained.

#### Cells clustering

Cells were clustered according to the Seurat v4.0.4 [27] Guided Clustering Tutorial. The first step is to remove low-quality cells, and the principle of retention: retain genes expressed in at least 3 cells, and retain cells with more than 200 genes and less than 6,000 genes detected and mitochondria accounting for less than 20%. After removing low-quality cells, the data were normalized using Seurat, and the top 2000 genes with high variability were selected for dimensionality reduction and clustering. Using a graph-based clustering algorithm, cells with close expression patterns are grouped into one group, and batch effects are removed [27], and then dimensionality reduction and clustering analysis are performed. Cells were organized into a total of 12 clusters. Non-linear dimensional reduction methods of UMAP were used to visualize the clustered cells.

#### Differential gene identification and marker gene identification

Based on the cell clustering results, differentially expressed genes between a certain cluster and other clusters were identified based on the expression of cells in each cluster. The screening principle of differentially expressed genes: at least 25% of cells are expressed, and in different groups, the proportion of expressing cells differs by more than 25%, and the logFC threshold for the fold change of differential expression is 0.25, and the significant adjust *p* value threshold is 0.01. Based on the graph-based clustering and difference results, the differentially expressed genes corresponding to each cluster were further screened, and the genes with the most significant differences and up-regulated expression patterns were selected as the marker genes of the cluster.

#### Cell type annotation

Using SingleR [28] software (v1.6.1), the cells in the test dataset were labeled and annotated similar to the reference set by given cell samples with known cell type labels as reference datasets (Blueprint/ENCODE projects).

#### Copy number variation estimation

Copy number variations (CNVs) with the infercnv (v1.10.1, https://github.com/broadinstitute/inferCNV/wiki). The extent of the CNV signal of each cell was scored, defined as the mean of squares of CNV values across the genome.

#### Function analysis

The clusterProfiler (v3.4.4) was used to analyse the relative activation of a given gene set such as the Kyoto Encyclopedia of Genes and Genomes (KEGG) pathways, Gene Ontology (GO) and Human Disease Ontology [29].

#### Ligand and receptor analysis

CellPhoneDB was used to analyse of the interactions of receptors and ligands. The normalised cell matrix was achieved by Seurat Normalization [30].

## Results

### Cell clustering of anaplastic ependymoma and H3K27M-mutant diffuse midline glioma

**Fig. 1:**
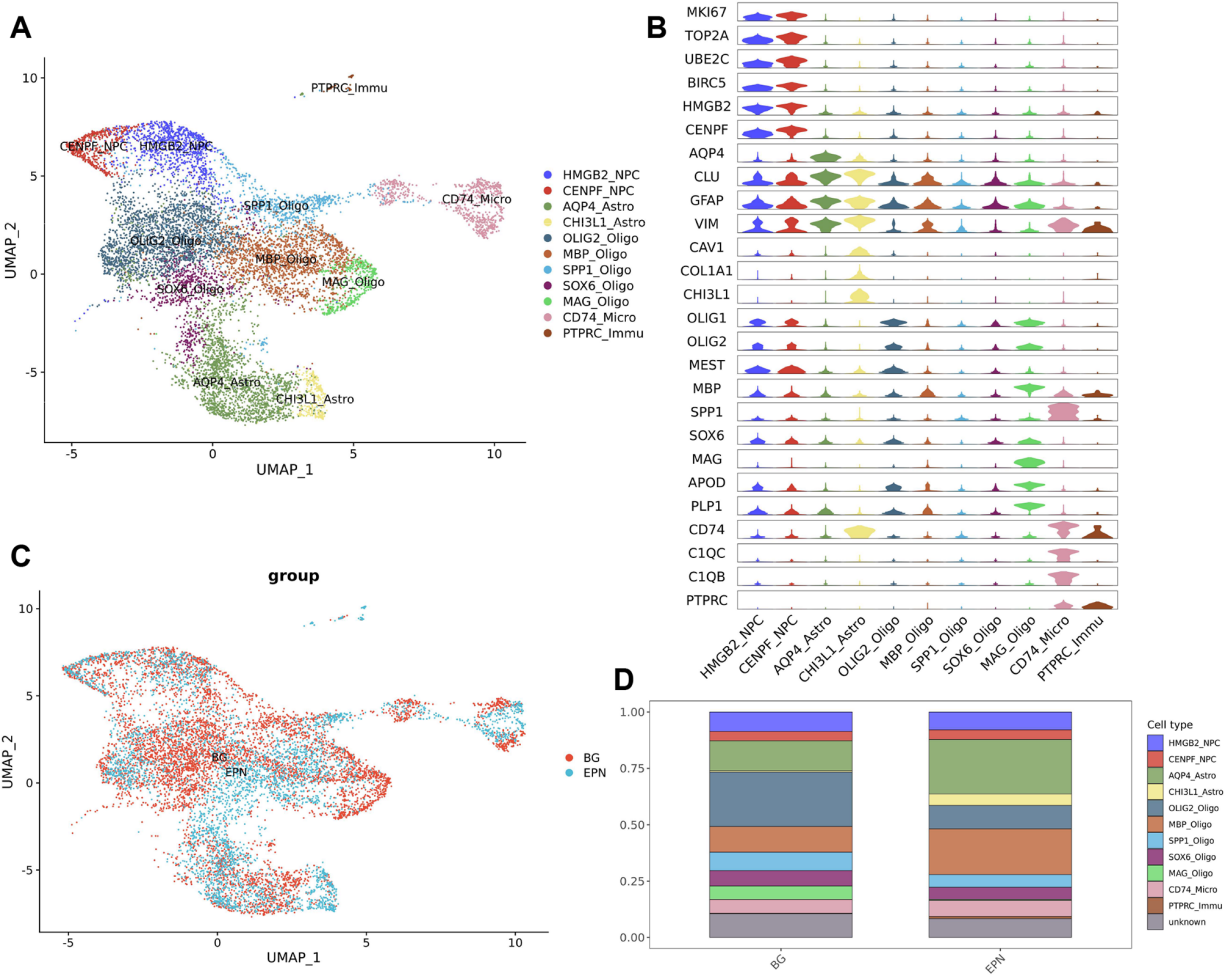
Comprehensive cellular overview of EPN and BG. **(A)** UMAP plots for the cell type identification of high-quality single cells. **(B)** Violin plots showing the expression of marker genes in distinct clusters. **(C)** The UMAP plots showing sample source of cell populations. **(D)** The histograms showed percentage of EPN and BG in different cluster

To investigate the cellular heterogeneity and molecular signatures in anaplastic ependymoma and H3K27M mutant diffuse midline glioma, we generated single cell RNA profiles from anaplastic ependymoma sample and H3K27M mutant diffuse midline glioma sample by 10× single-cell RNA-seq. The clinical characteristics of these patients are presented in Table S1. Following filtering and gene expression normalization by Cell Ranger (10× Genomics) and Seurat analysis [31], we performed uniform manifold approximation and projection of cells to identify different cell clusters. After gene expression normalization, 11,219 cells were retained for further analysis, of which 6,998 were from BG and 4,221 from EPN. Based on cell-specific markers and significantly enriched genes, cells were categorized into 12 clusters, including 2 astrocytes clusters (AQP4+, CLU+, GFAP + cluster and VIM+, CLU+, CHI3L1+, COL1A1 + cluster), oligodendrocytes (OLIG1+, OLIG2+, MEST + cluster, MBP+, PLP + cluster, OLIG1+, OLIG2+, SPP1 + cluster, OLIG1+, OLIG2+, SOX6 + cluster, MAG+, APOD+, PLP1 + cluster), neural progenitor cell (HMGB2+, MKI67+, TOP2A+, UBE2C + cluster and BIRC5+, MKI67+, TOP2A+, UBE2C + cluster), microglial cell (CD74+, C1QC+, C1QB+), immune cells (PTPRC+) and an unknown cluster **(**Fig. 1A **and B)**. In the BG sample, the OLIG2_Oligo cluster had the largest cell proportion (24.14%), whereas the AQP4_Astro cluster (24.12%) was the largest subcluster in the EPN sample **(**Fig. 1C-D**)**. This is consistent with the classification of cell clusters of gliomas obtained by the identification of Filbin et al [32].

**Fig. 2:**
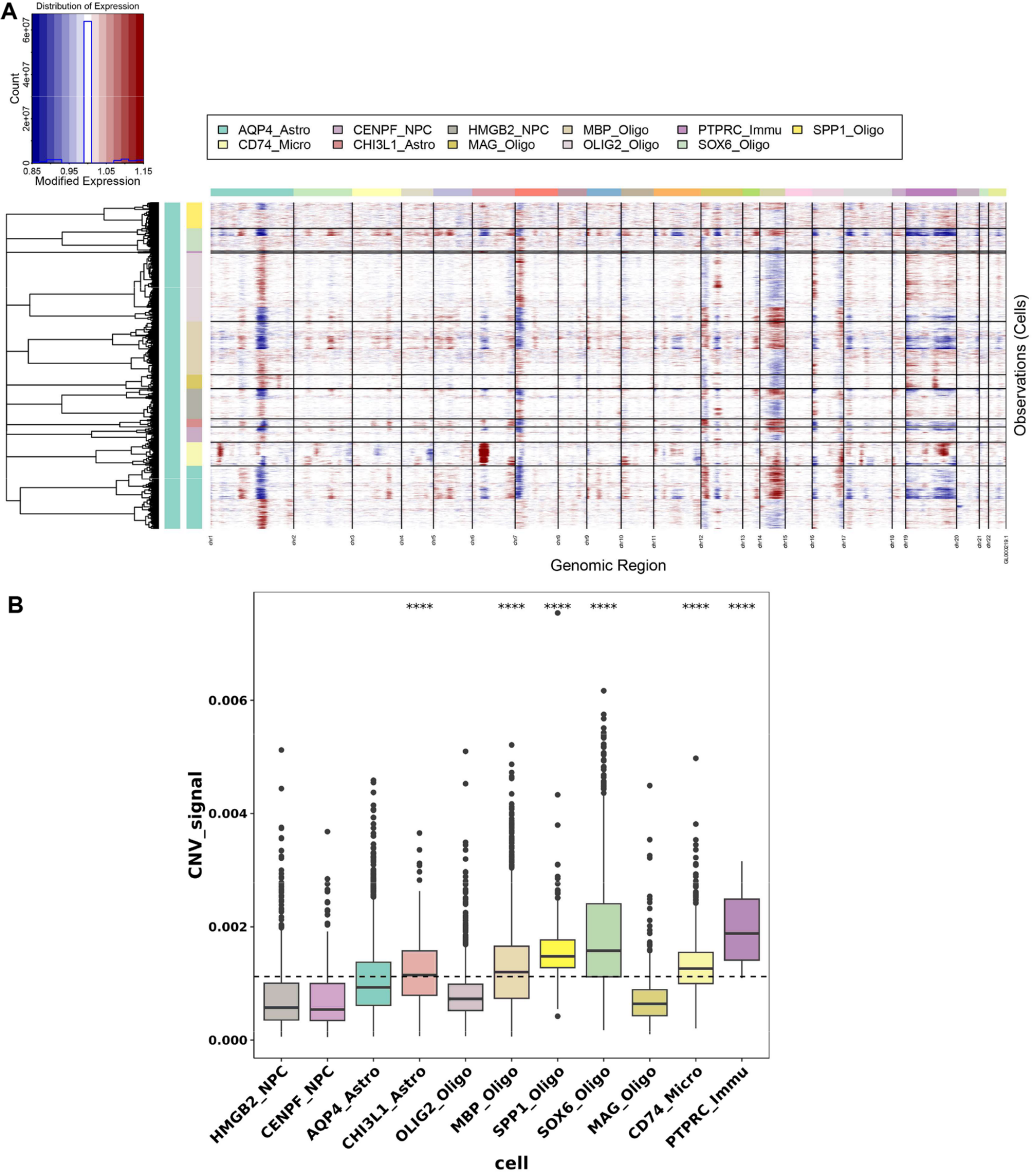
Analysis of distinct cell clusters. **(A-B)** CNV heatmap and box plots for 11 distinct cell clusters

Moreover, multiple copy number variations (CNVs) were found on different chromosomes in two tumor subtypes (Fig. 2A). The lowest CNV score were found in neural progenitor cells, while the highest were found in SOX6_Oligo and PTPRC immune clusters, implying that these clusters may be malignant cells (Fig. 2B).

### Transcriptomic inter-tumor heterogeneity of astrocytes

**Fig. 3:**
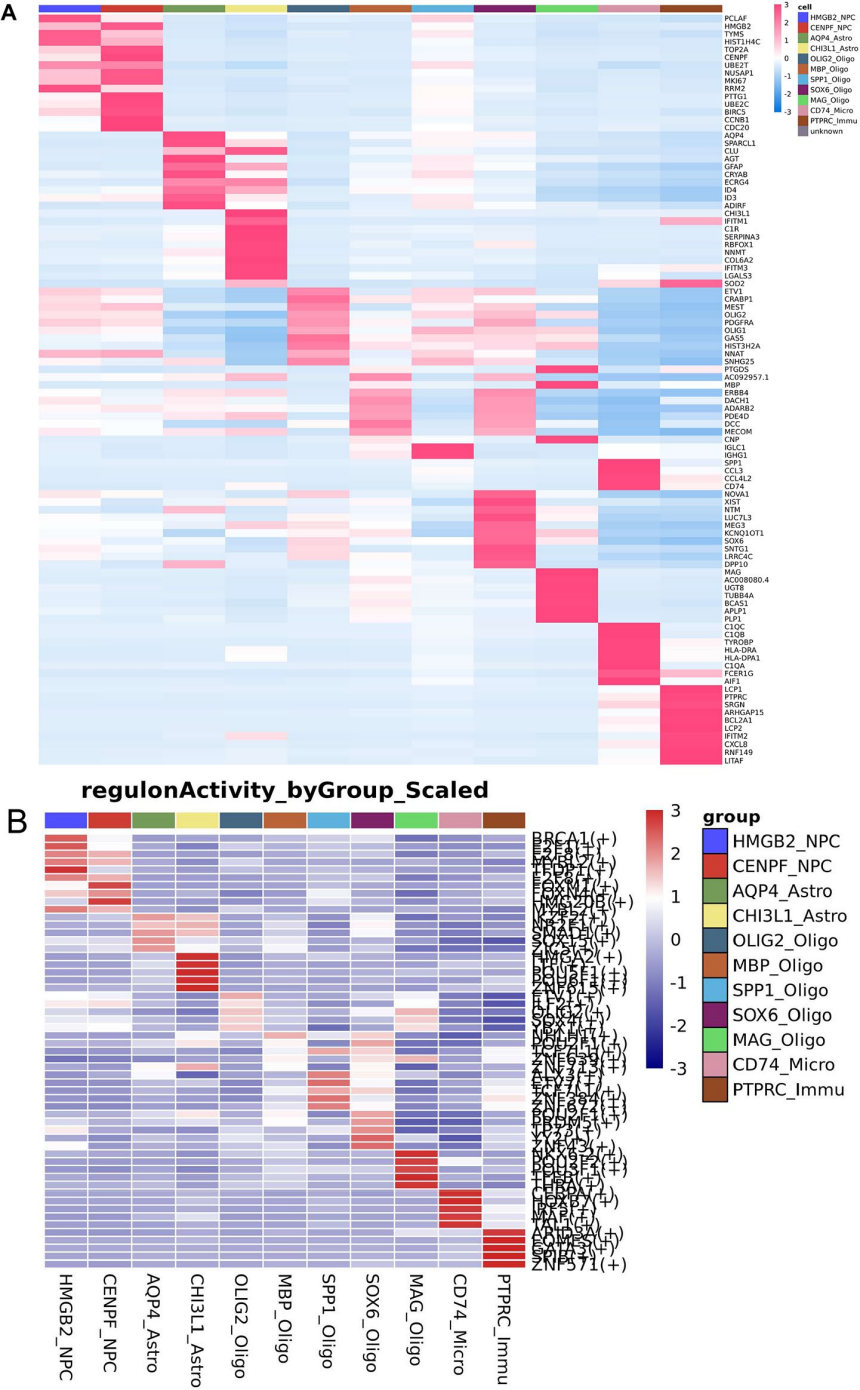
Heatmap of distinct cell clusters. **(A)** Heatmap of top genes in distinct cell clusters. **(B)** SCENIC analysis showed activated transcription factors in distinct cell clusters

**Fig. 4:**
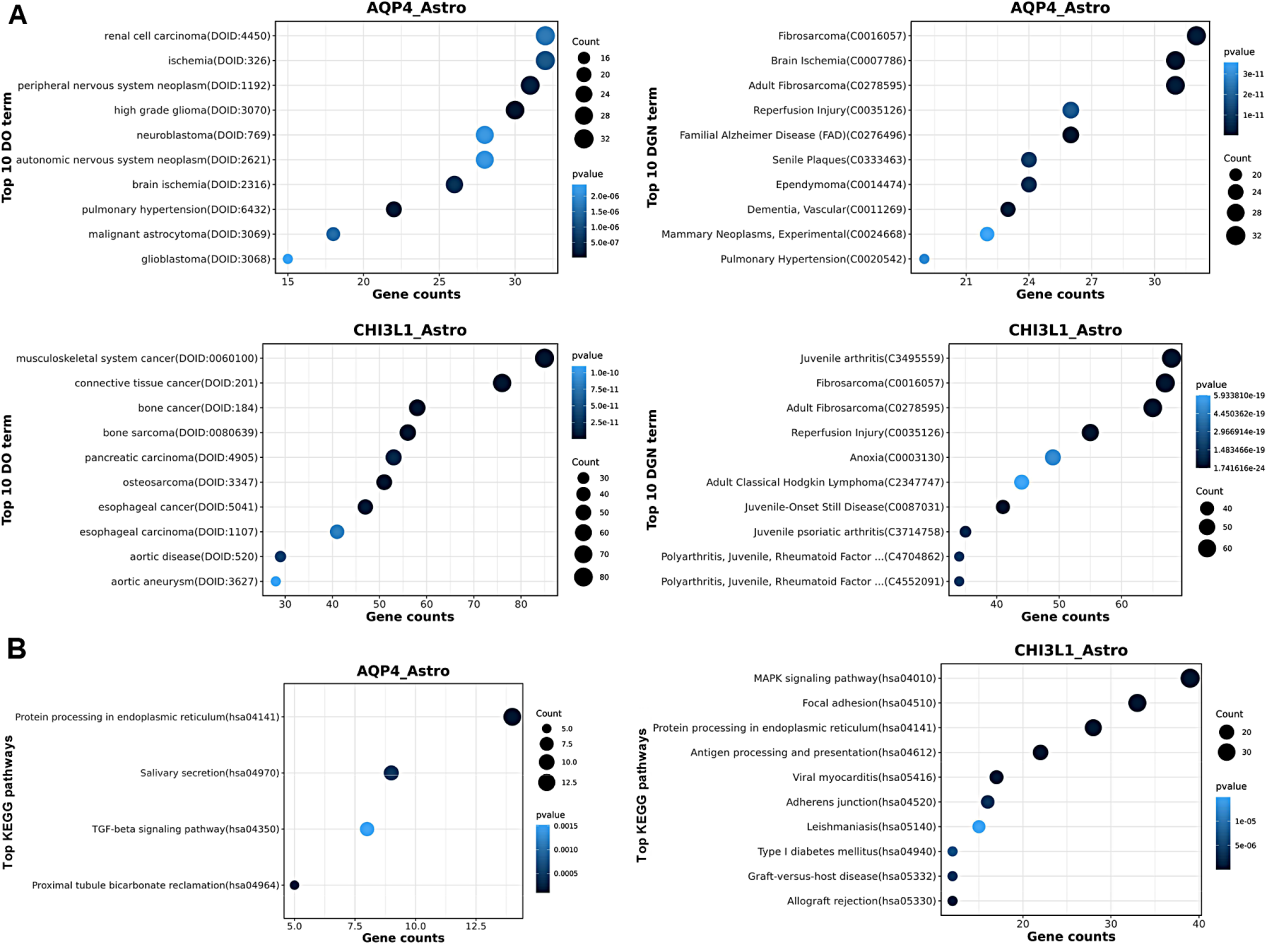
Function analysis of DISEASE **(A)** and KEGG **(B)** in two astrocyte clusters

Astrocytes were categorized into AQP4_Astrocytes and CHI3L1_Astrocytes cluster. The AQP4_Astrocytes expressed high levels of CLU, AQP4, SPARCL1, AGT, GFAP, CRYAB, and CRABP1 (Fig. 3A). Function analysis of Disease Ontology showed that the cluster were associated with various cancers, such as neuroblastoma, glioma and glioblastoma (Fig. 4A). KEGG analysis indicated some classical cancer-related pathways were enriched, such as Wnt pathway, MAPK pathway and TGF-beta pathway (Fig. 4B). SCENIC method identified the top 5 underlying transcription factors in AQP4_Astrocytes cluster were IKZF2, NR2F1, SMAD1, SOX15, and ZIC5 (Fig. 3B). And transcription factors HMGA2, LTF, POU5F1, POU6F1 and ZNF615 were enriched in CHI3L1_Astrocytes cluster (Fig. 3B).

### Transcriptomic inter-tumor heterogeneity of oligodendrocyte

**Fig. 5:**
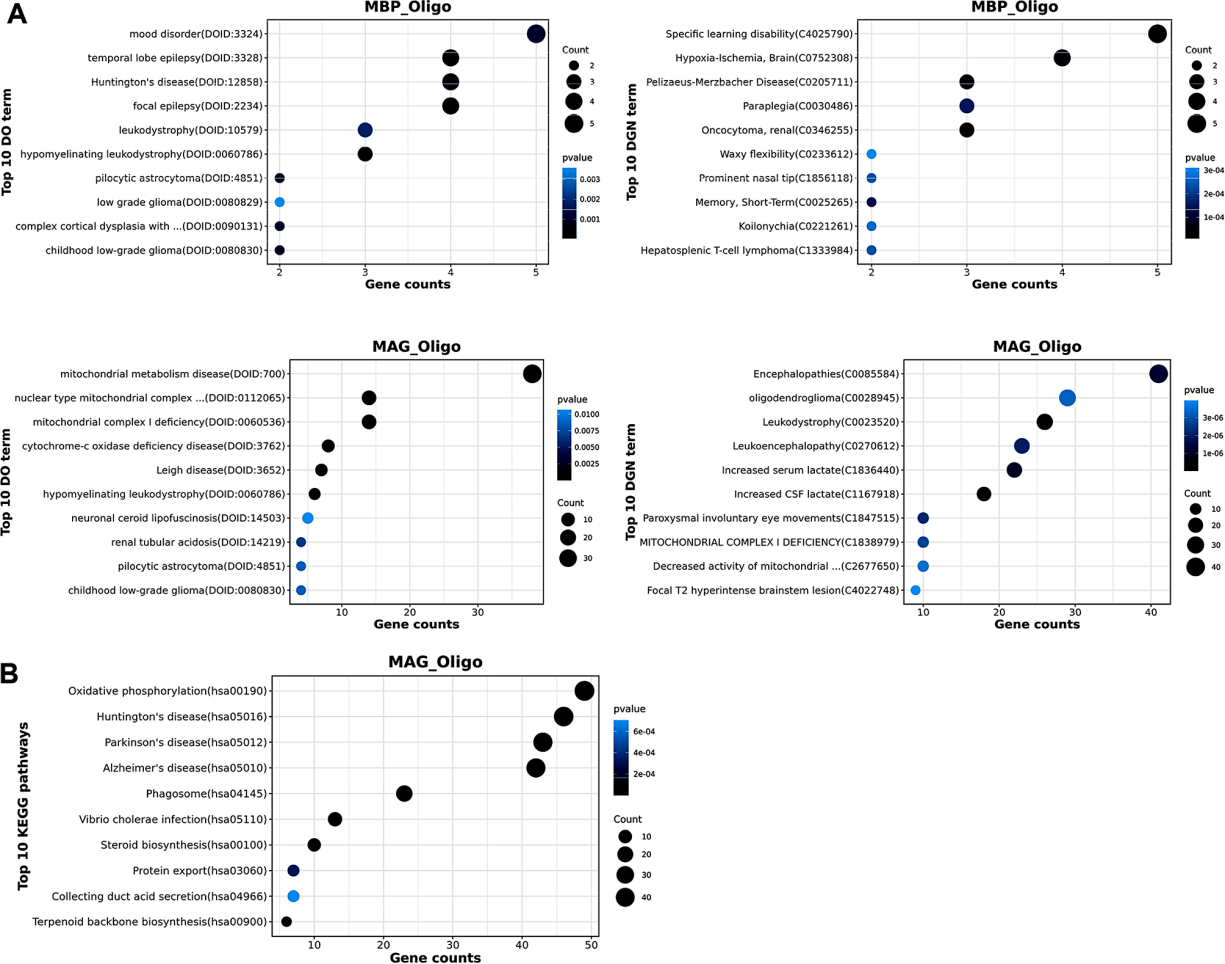
Function analysis of DISEASE **(A)** and KEGG **(B)** in two oligodendrocyte clusters

The oligodendrocyte cells exhibited 5 distinct subclusters, labeled OLIG2_Oligo (CRABP1, ETV1, OLIG1, OLIG2, MEST), MBP_Oligo (PTGDS, MBP, ADARB2, ERBB4, DACH1), SPP1_Oligo (IGLC1, IGHG1, SPP1, CCL3, CCL4L2), SOX6_Oligo (NTM, NOVA1, XIST, MEG3, KCNQ10T1), MAG_Oligo (MAG, MBP, PTGDS, PLP1, TUBB4A) (Fig. 3A). Disease Ontology analysis showed that MAG_Oligo and MBP_Oligo were highly associated with cancer (Fig. 5A). KEGG analysis indicated genes of MAG_Oligo and MBP_Oligo are enriched in neurological disease signal, such as huntington’s disease, parkinson’s disease (Fig. 5B). Transcription factors such as NKX6-2, POU2F2, POU3F1, TFEB, THRA were enriched in MAG_Oligo, while NHLH1, POU2F1, TCF7L1, ZNF639, ZNF713 were enriched in MBP_Oligo (Fig. 3B).

### Transcriptomic inter-tumor heterogeneity of microgliacyte

**Fig. 6:**
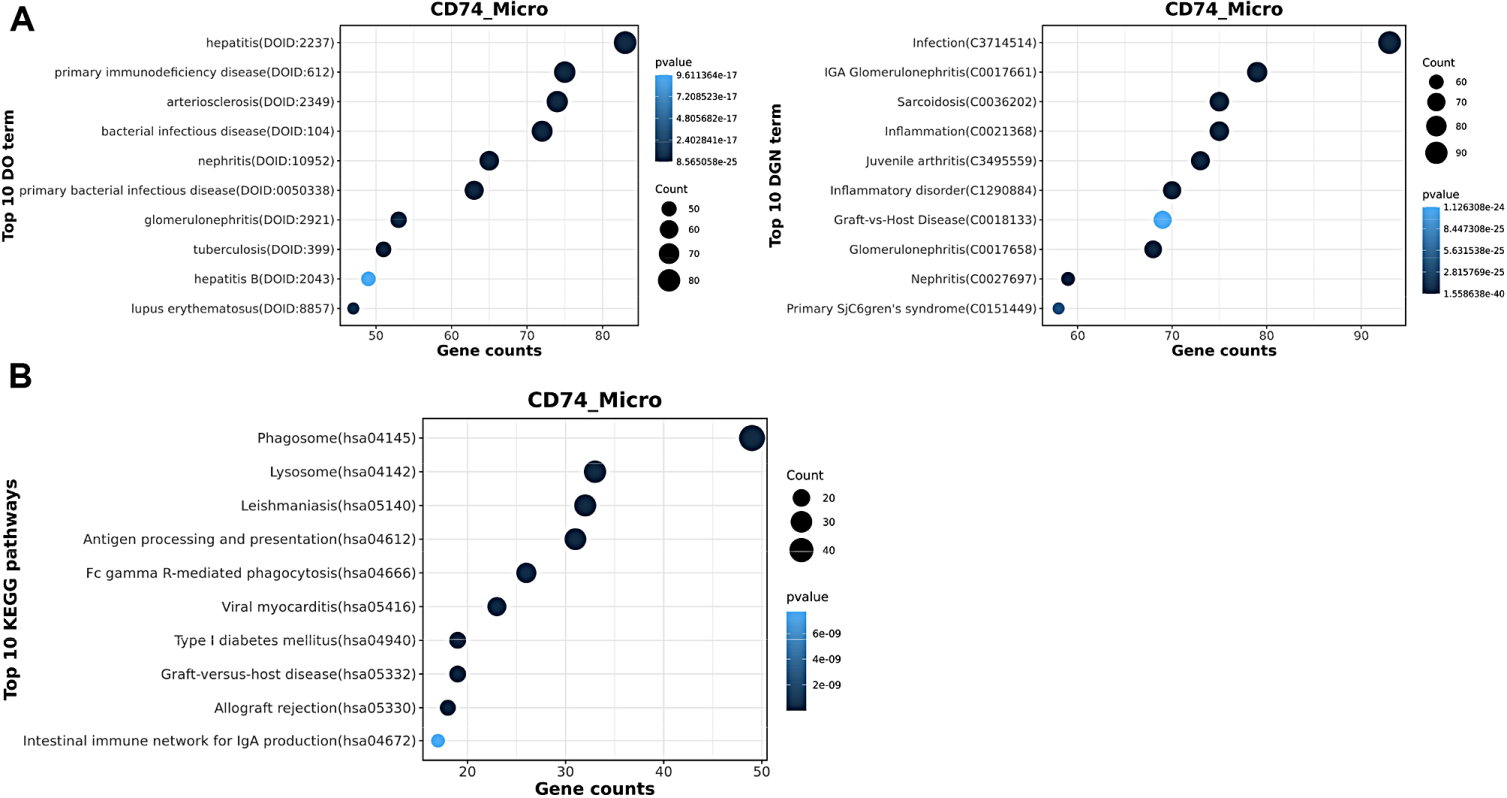
Function analysis of DISEASE **(A)** and KEGG **(B)** in microglial cell clusters

A unique microgliacyte cluster, CD74_ micro (Top 5 genes CD74, C1QC, C1QB, TYROBP, HLA-DRA), was observed (Fig. 3A). The Disease Ontology analysis showed all highly associated with cancer pathway (Fig. 6A). KEGG results showed MAPK pathway, ErbB pathway and T cell receptor signaling pathway were enriched (Fig. 6B). Transcription factors CEBPA, HOXB7, IRF5, MAF, TAL1 were enriched in CD74_ micro cluster (Fig. 3B). The expression patterns of these genes may reflect subtle differences between the cells of origin [32].

### Cell communication inter-tumor

**Fig. 7:**
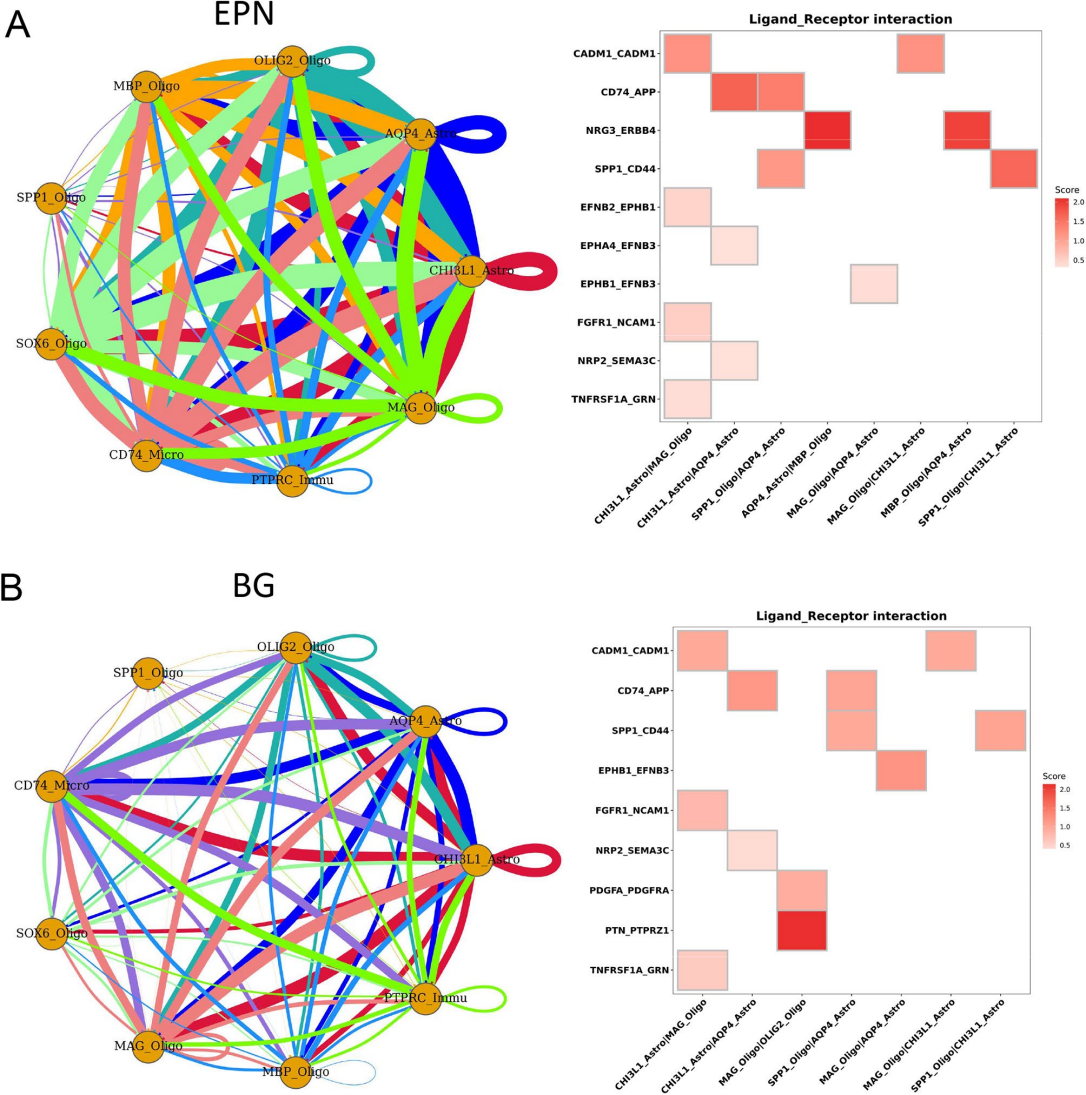
Interaction analysis. **(A)** Interaction analysis showing enriched receptor-ligand pairs in cell cluster derived from EPN. **(B)** Interaction analysis showing enriched receptor-ligand pairs in cell cluster derived from BG

Ligand-receptor analysis was used to predict intercellular communication between different cell clusters. The cell clusters of EPN had more gene interaction pair than the cell clusters of BG (Fig. 7A). In the EPN group, the NRG3-ERBB4 pair was enriched in the interactions between AQP4_Astro and MBP_Oligo. In the BG group, the PTN-PTPRZ1 pair was most enriched pair in MAG_Oligo and OLIG2_Oligo (Fig. 7B). The results indicate that astrocytes, oligodendrocytes, and microglial cells establish contact with other cells through NRG3-ERBB4 and PTN-PTPRZ1 ligand-receptor pairs.

## Discussion

Brain tumors are the most common solid tumors in children, cause severe morbidity and mortality worldwide [33]. Current research suggests that the occurrence of brain tumors is related to genetic and environmental factors [33, 34]. However, the pathogenesis of different subtypes of brain tumors is different, and so are their tumor immune microenvironment and signaling molecules. Anaplastic ependymoma and H3K27M-mutant diffuse midline glioma are two common subtypes of brain tumors with suboptimal long-term prognosis [1]. These two types of brain tumors are susceptible to resistance to chemotherapy, and no suitable targeted therapy agents are currently available. Single-cell RNA sequencing technology can analyze complex tissue transcriptomes at the single-cell level, allowing for a comprehensive and accurate research on cell types and molecular signals in tumor tissue [35, 36].

In this study, we performed single-cell sequencing of anaplastic ependymoma and H3K27M-mutant diffuse midline glioma, and analyze the cellular composition, differentially expressed genes and signaling pathways. We first remove the batch effect and then perform dimensionality reduction and cluster analysis, finally identifying 12 clusters. The AQP4_Astro cluster accounted for the largest proportion in anaplastic ependymoma, while OLIG2_oligo was the largest cluster in H3K27M-mutant diffuse midline glioma. The discordance of the cell population types and proportions between the two brain tumors suggested that two tumor types had different tumor microenvironments, further illustrating the difference in the tumor microenvironment of these two tumors. According to our analysis, all cells can be classified into 11 clusters, with their own unique cell marker. Both EPN and BG had high population of AQP4_Astro cluster with marker genes CLU and AQP4, which are malignant metastasis biomarker. The AQP4_Astro cluster may represent some common features between EPN and BG. Evidence has demonstrated that AQP4, which is usually highly expressed in brain tissues, promotes the tendency of tumor-associated macrophages to become M1 macrophages, thereby causing alterations in the cellular state of gliomas [37, 38]. Increased expression of AQP4 leads to a reduced OS in malignant gliomas, resulting in a poor prognosis [37]. Therefore, it is necessary to further explore reliable biomarkers and develop suitable targeted therapy methods based on intratumor heterogeneity.

We found AQP4_Astrocytes was enriched in Wnt pathway, MAPK pathway and TGF-beta pathway. Activation of Wnt signal is a feature of Medulloblastomas, and embryonic radial glial cells in the neocortex expressing AQP4 are essential for vascular and blood-brain barrier formation by regulating the Wnt signaling pathway [39, 40]. Ryall et al., reported Pediatric low-grade gliomas were frequently driven by genetic alterations in RAS/MAPK pathway [41]. Human erythropoietin protects astrocytes from swelling induced by ischemia and reperfusion-like injury, and this neuroprotective capacity is mediated by reducing MAPK activity-dependent excess of AQP4 in plasma membrane [40]. TGF-β pathway is a potentially useful therapeutic target in glioblastoma multiforme [42]. It has been reported that any central nervous system disease characterized by upregulation of AQP4 on astrocytes may also involve the AQP4/TGFB1 pathway [43]. These studies further demonstrate the validity and reliability of the results of the present study.

Interestingly, AQP4_Astrocytes cluster derived from EPN highly expressed some anti-cancer genes, such as JUN, IRF1 and MSX1. MSX1 was a biomarker for the prognosis of colorectal cancer and a transcription factor involved in neural crest development that synergistically promotes astrocyte differentiation with other family members [43, 44]. IRF1 is a crucial regulator, the inflammation-driven activity of IRF1 and NF-κB promotes the reactivation of endogenous retrovirus K in motor cortical neurons of amyotrophic lateral sclerosis, and may play a role in the resistance of glioma to immune checkpoint blockade [44, 45]. These studies further suggest that AQP4_Astrocytes exert vital regulatory roles in EPN and may be a key cell population in the treatment of tumors.

Furthermore, we found that astrocytes, oligodendrocytes, and microglial cells associate with other cells through NRG3-ERBB4 and PTN-PTPRZ1 ligand-receptor pairs. Ablation of NRG3 and ERBB4 leads to a reduction in the number of excitatory synapses on small parvalbumin-positive interneurons, altered short-term neuroplasticity, and disinhibition of the hippocampal network, which plays a critical role in intercellular communication in alzheimer’s disease [46]. NRG3/ERBB4 signaling is a therapeutic target for canine glioma [47]. Tumor-associated macrophages secrete abundant pleiotrophin (PTN) to stimulate glioma stem cells via their receptor PTPRZ1, thereby promoting malignant growth of glioblastoma through PTN-PTPRZ1 paracrine signaling [48]. The tumor microenvironment is highly enriched for immunosuppressed M2-like tumor-associated macrophages and glioblastoma stem cells, which promote glioblastoma malignancy through the PTN-PTPRZ1 signaling axis [49]. Further studies targeting NRG3-ERBB4 and PTN-PTPRZ1 ligand-receptor pairs will help to provide a new indicative strategy for the treatment of gliomas.

## Conclusion

In this study, we analyzed H3K27M-mutant diffuse midline gliomas and anaplastic ependymomas by single-cell RNA sequencing technology, and found that there was intratumoral heterogeneity in both tumors. Differentially expressed mRNAs from different clusters can be used for the differential diagnosis of these two tumors and may be potential therapeutic targets. Further analyses combining comparison of tumor and cancer peripheral tissue may obtain more promising evidence. Nevertheless, the existing data can still show a comprehensive understanding and insight into these two tumors as a whole. These data can be used to formulate more strategies for the prevention and treatment of brain tumors in the future.

### Electronic supplementary material

Below is the link to the electronic supplementary material.

Supplementary Material 1: Clinical information of samples

## Data Availability

The original data set generated and/or analyzed during the current study can be obtained from GEO [GSE245674].
