# Supplementary material for: Single-cell RNA sequencing of anaplastic ependymoma and H3K27M-mutant diffuse midline glioma
Source: BMC Neurol. 2024 Feb 21;24:74. doi: 10.1186/s12883-024-03558-7 (PMC10880286; doi:10.1186/s12883-024-03558-7)
Supplement: Supplementary file 1 — Supplementary Material 1: Clinical information of samples [file 12883_2024_3558_MOESM1_ESM.docx]

Supplementary Table 1. Clinical information of samples

| Item | Samples 1 | Samples 2 |
| --- | --- | --- |
| Sex | female | female |
| Age | 10y | 2y |
| Clinical symptoms | Unsteady gait, dyskinesia | headache, vomiting |
| Sampling site | Brain stem, pontine cerebellum mass; gray-white soft tissue | cerebellopontine angle, fourth ventricle |
| Visual observation of tumor | brain stem | Intracranial mass, a pile of gray-red gray-white soft tissue |
| light microscope | The tumors in the samples were diffusely infiltrating growth with varying density, round or oval nuclei of tumor cells, easy to see mitoses, obvious proliferation of interstitial blood vessels, and some of them were glomerular-like. | Tumor cells are arranged in pseudo rosette-like clusters around blood vessels, with increased layers of tumor cells arranged around blood vessels, a few vascular endothelial hyperplasia, increased cell density, hyperchromatic nuclei, round or oval shape, mitoses, obvious interstitial hemorrhage, and localized Necrosis can be seen in the foci. |
| Pathological diagnosis | Diffuse midline glioma, H3K27M-mut ant, WHO grade Ⅳ | Anaplastic ependymoma, WHO grade III |
| Immunohistochemistry | GFAP（+） | H3K27M（-），GFAP（+） |
